# Silver Nanoparticles on Chitosan/Silica Nanofibers: Characterization and Antibacterial Activity

**DOI:** 10.3390/ijms21010166

**Published:** 2019-12-25

**Authors:** Małgorzata Zienkiewicz-Strzałka, Anna Deryło-Marczewska, Yury A. Skorik, Valentina A. Petrova, Adam Choma, Iwona Komaniecka

**Affiliations:** 1 Department of Physical Chemistry, Institute of Chemical Sciences, Maria Curie-Skłodowska University, sq. Maria Curie-Skłodowska 3, 20-031 Lublin, Poland; annad@hektor.umcs.lublin.pl; 2 Institute of Macromolecular Compounds, Russian Academy of Sciences, Bolshoi pr. VO 31, St. Petersburg 199004, Russia; valentina_petrova_49@mail.ru; 3 Department of Genetics and Microbiology, Maria Curie-Sklodowska University, Akademicka 19, 20-033 Lublin, Poland; adam.choma@poczta.umcs.lublin.pl (A.C.); ikoma@hektor.umcs.lublin.pl (I.K.)

**Keywords:** chitosan nanofiber, nanocomposite, silver nanoparticles, antibacterial activity, electrospinning

## Abstract

A simple, low-cost, and reproducible method for creating materials with even silver nanoparticles (AgNP) dispersion was established. Chitosan nanofibers with silica phase (CS/silica) were synthesized by an electrospinning technique to obtain highly porous 3D nanofiber scaffolds. Silver nanoparticles in the form of a well-dispersed metallic phase were synthesized in an external preparation step and embedded in the CS/silica nanofibers by deposition for obtaining chitosan nanofibers with silica phase decorated by silver nanoparticles (Ag/CS/silica). The antibacterial activity of investigated materials was tested using Gram-positive and Gram-negative bacteria. The results were compared with the properties of the nanocomposite without silver nanoparticles and a colloidal solution of AgNP. The minimum inhibitory concentration (MIC) of obtained AgNP against *Staphylococcus aureus* (*S. aureus*) ATCC25923 and *Escherichia coli* (*E. coli*) ATCC25922 was determined. The physicochemical characterization of Ag/CS/silica nanofibers using various analytical techniques, as well as the applicability of these techniques in the characterization of this type of nanocomposite, is presented. The resulting Ag/CS/silica nanocomposites (Ag/CS/silica nanofibers) were characterized by small angle X-ray scattering (SAXS), X-ray diffraction (XRD), and atomic force microscopy (AFM). The morphology of the AgNP in solution, both initial and extracted from composite, the properties of composites, the size, and crystallinity of the nanoparticles, and the characteristics of the chitosan fibers were determined by electron microscopy (SEM and TEM).

## 1. Introduction

Hybrid nanocomposites are an important platform for the preparation of new materials with advanced properties [1,2,3,4,5,6,7,8]. Noble metal nanostructures encapsulated within or deposited on biopolymers are of particular interest for human [9,10], sensing [11,12], and catalytic applications [13,14,15,16], as these types of materials combine the physicochemical features of noble metal nanostructures and the biological features of polymers [17,18]. In this context, chitosan-based nanocomposites are becoming increasingly important due to their multi-functionality, biocompatibility, biodegradability, wide-ranging pH stability, and activity as bioadsorbents [19,20,21,22,23,24,25].

**Scheme 1:**
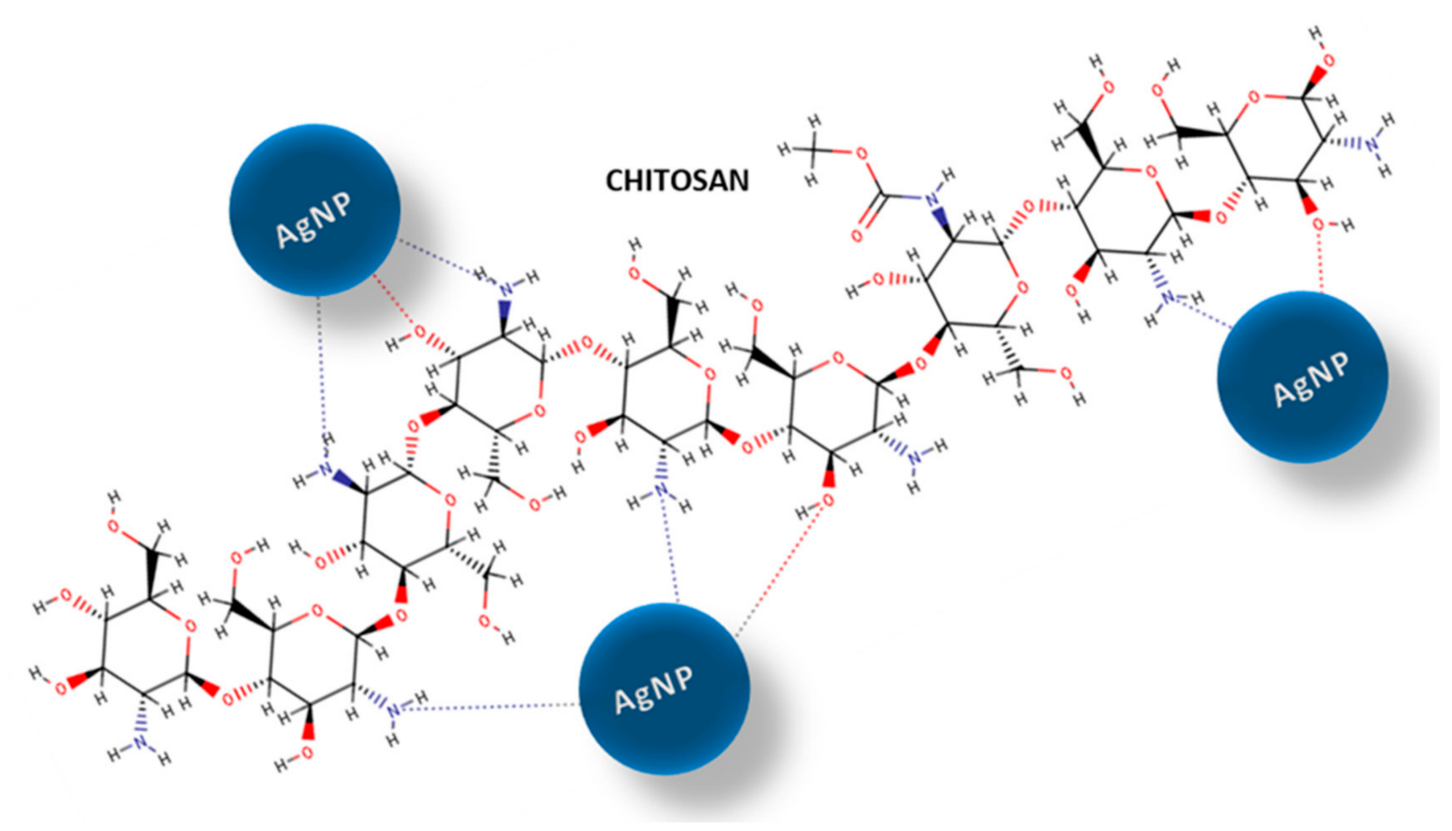
Schematic diagram of showing interaction of chitosan matrix with silver nanoparticles (AgNPs). The amine and hydroxyl groups in chitosan structure are responsible for possible interaction and stabilization of metallic phase.

The chitosan structure contains unique functional groups that play an important role in chitosan interactions with metal ions and metal nanoparticles. In particular, the primary amino groups of chitosan interact with metal surfaces and also act as capping sites for the nanoparticle stabilization [26,27]. Consequently, chitosan has the ability to form composites with silver and other noble metals in their ionic or metallic forms. The chitosan-metal interactions probably reflect the coordination of metal ions via the amino groups present on the biopolymer chain [28]. The formation of nanoparticle–polymer-coordinated complexes can also reflect the number of free amine functions on the surface and the hydrophilic properties of the adsorbent macromolecules [29]. Furthermore, the reduction of silver ions can occur by oxidation of other functional groups on the chitosan structure (e.g., hydroxyl groups). One possible explanation for the affinity of chitosan for silver, as well as for other metal nanoparticles, could be the creation of chemical bonds between the nitrogen atoms, which are considered electron-rich elements, and the lone pairs in the silver orbitals. These properties are particularly important in the formation of new functional systems. The stability of metal nanoparticles on solid surfaces is a significant factor determining the antibacterial efficacy of new nanocomposites. The proposed interaction model between chitosan matrix and AgNP is shown in Scheme 1.

The influence and interaction of noble metal nanoparticles (for example, AgNP) with bacteria are more substantial when the nanoparticles are very small and well dispersed on the carrier surface [30]. The role of chitosan in a biocomposite, therefore, does not have to be limited to its role as a matrix. Chitosan can act as a stabilizing agent for metal nanoparticles by forming a network on the surface of other supports, such as carbon nanotubes. This type of composite has been applied as a carbon electrode and showed substantial electron transfer behavior [31]. The stabilizing features of chitosan have been tested for green and simple procedures of silver-chitosan nanocomposite preparation using AgNO_3_ as a silver nanoparticle precursor and glucose as a reducing agent [32]. Chitosan also has known antibacterial effects on its own, as the amine groups of chitosan can react with the anionic groups on the surfaces of bacteria cells and alter bacterial cell permeability. These properties of nanocomposites based on chitosan are responsible for the growing interest in these materials and fully justify the ongoing research in this area.

According to the literature reports, a silica phase can be incorporated into the chitosan matrix to improve the mechanical properties of a biopolymer [33]. The creation of inorganic/biopolymer composites as chitosan/silica hybrids with advanced physicochemical properties determines the extent of the improvement of biopolymer stability by the bioinert component. The presented research examines the possibility of introducing a silica phase into a biopolymer structure without generating a separated siliceous phase in the form of silica layers or agglomerates [34]. This is one of the best ways to synthesize homogeneous composites. Additionally, the nanofiber forms of composites that are useful in practice have many advantages, including large and active surfaces and forms that are desirable for use in sensors and as affinity membranes (e.g., air filtration membranes, water filtration membranes, scaffolds for tissue engineering or wound care patches) [35,36,37]. Moreover, the usefulness of this type of composite in the form of nanofiber sheets is growing when compared to powders, gel systems, and others. Composites in nanofiber form serve to improve the tunable and mechanical properties, the tailorable degree of porosity, and the external porous architecture, as well as the controlled degradability. The 3D scaffolds in nanofiber form ensure an enhanced surface for metallic nanoparticles. The attachment of metal nanoparticles onto nanofibers may be a way of inducing metal nanoparticles to form macroscopic 3D structures. The use of nanofibers in supports also has a great advantage from the point of view of nanoparticle use applications, as it allows homogeneous nanoparticle distribution on the surface, with no visible aggregation effects. This, in turn, determines the biological usefulness of the nanoparticles, as well as their bioavailability, by covering the external surface of the carrier—a coverage that is difficult to achieve when using powders and highly porous materials [38]. Biopolymeric nanocomposites decorated with a metallic nanophase are advanced functional materials. The design and preparation of these advanced systems have one main purpose—the improvement and stabilization of the biostatic or antibacterial properties [39,40,41,42]. However, the cytotoxicity of the nanocomposites should also be considered. In the case of chitosan nanofibers, the fabrication (at the macro scale) of biocompatible and non-toxic materials is realized. The chemistry of the chitosan surface (active functional groups) lends itself to the selective interaction of an active agent with bacterial cells and an affinity for AgNP; therefore, Ag/CS nanocomposites seem to be perfect resources for the preparation of functional biomaterials.

The general aim of this work was to investigate the preparation of new composites and the immobilization of silver nanoparticles on their surfaces and to describe the structural and surface properties and confirmation the activity of these biopolymer-decorated metal nanoparticles against Gram-positive (*Staphylococcus aureus* ATCC25923), and the Gram-negative (*Escherichia coli* ATCC25922) bacteria. In this work, the additional silica phase wad added for building more stable nanofibers architecture. The applied silica phases include fumed silica materials with the particle size of 0.2 µm and 0.007 µm marked as SiO_2__02 and SiO_2__007 respectively as well as mesoporous ordered silica phase marked as SBA-15.

In many studies, chitosan in its various forms is considered bactericidal [43,44], but these properties have recently been limited to biostatic ones [45,46,47,48]. The research carried out in the present work will also be useful in explaining this phenomenon. The results show convincingly that AgNPs were homogenously and finely dispersed over the whole biopolymer matrix without the use of additional capping agents while preserving their own fine dimensions. Moreover, their attachment to the biopolymer matrix was sufficiently strong to retain the metallic silver nanoparticles on the surface without release to the outside environment.

## 2. Results and Discussion

### 2.1. Characterization of the AgNP solution

**Figure 1:**
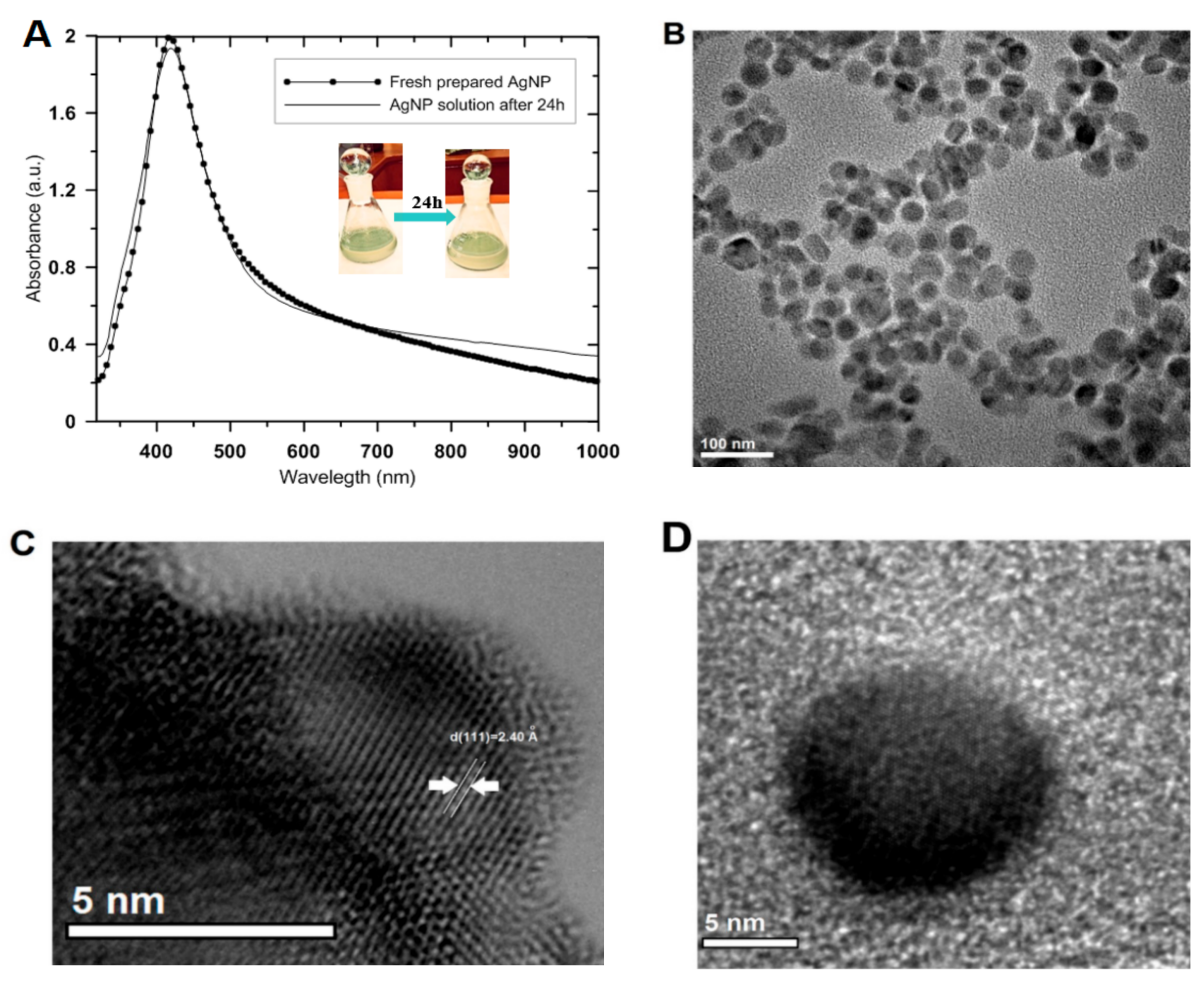
(**A**) UV-Vis absorption spectra of silver nanoparticle colloidal solutions (freshly prepared and after storage for 24 h) and digital photos of experimental probes of AgNP. (**B**) Transmission electron microscope images of the colloidal silver solution. (**C**,**D**) High-resolution TEM images of silver nanocrystals. Note: The concentration of the colloidal silver solution was adjusted by dilution in distilled water for the best visibility in the UV-Vis spectrum (maximum absorbance ~2).

The silver concentration of the initial AgNP solution was determined as 43.5 mg/L by atomic absorption spectrometry. At this concentration, the final solution of silver nanoparticles showed good quality, as confirmed by their spectral characteristics. It is important to obtain and confirm a high yield and quality of the synthesized nanoparticles, which can be used for clinical and commercial purposes. The high quality of the prepared AgNP solution is associated with the narrow size distributions as well as clear and long-term stability, which was confirmed by the surface plasmon resonance characteristics and scattering across the visible and near-IR regions of the spectrum (Figure 1A). The spectral characteristics (intensity and half-width of the plasmonic peak) did not change significantly over time, suggesting maintenance of system stability. The UV-Vis absorption spectra were used to monitor the formation and to confirm the stability of AgNP during its deposition onto the solid surface. The stability and quality of the colloid system of nanoparticles were evaluated for 24 h to take into account the time required for experimental deposition on the nanofiber surface. The freshly prepared colloidal solution of silver nanoparticles generated an absorption band in the UV-Vis spectrum that illustrated a collective oscillation of electrons of the metal nanoparticles and resonance with the electromagnetic wave [49]. In this case, the nanocrystal dispersions generated an absorption/extinction peak at 420 nm.

Analysis of the solution quality at the time of deposition eliminated doubts regarding possible changes occurring in the nanoparticle solution itself. The obtained results suggest that the colloids were stable and suitable for deposition. No significant changes were noted in the size of the nanoparticles or the degree of agglomeration over a long period (4 weeks). TEM micrographs showed a mainly spherical shape for the particles, with sizes of 20–30 nm, with good dispersion in the stabilizer solution (Figure 1B), and with good crystal quality (visible lattice planes) (Figure 1C,D).

#### 2.1.1. Microstructural characterization of Ag/CS/silica nanofibers

Preparation of composites with the desired properties requires the addition of specific nano-additives. Particularly important characteristics include the shape and size of the nanoadditive particles, the degree of development of the surface, the crystalline nature, the specific surface area, the surface energy, and the method of the spatial distribution of nanoparticles in the polymer matrix. The silver concentration in the solution after deposition of AgNP onto the composite surfaces was determined again by atomic absorption spectrometry to test the deposition efficiency. The obtained results suggest that the concentration of silver in the AgNP solution decreased by approximately 46% (the silver concentration was 23.5 mg/L for the residual solution after deposition of the initial AgNP solution on the CS/SBA-15 composite). The nanoadditive phase on the Ag/CS/silica nanofibers was characterized using X-ray diffraction, which relies mainly on the positions of the diffraction peaks in the diffraction profiles. In this case, the crystalline nature of the silver nanoparticles was confirmed by the presence of well-defined and intense reflections of the silver metallic phase.

The wide-angle XRD patterns recorded for nanocomposites revealed the presence of intense diffraction peaks at at 2θ 38.1°, 44.3°, 64.5°, and 77.5°, which corresponded to distinct reflections for the Ag(111), Ag(200), Ag(220), and Ag(311) lattice planes of the cubic structure, respectively, and indicated good purity of the metallic silver phase (Joint Committee on Powder Diffraction Standards - JCPDS, File No. 04-0783). The lattice constant of the silver phase was calculated and the obtained values (*a* = 4.091 Å, *a* = 4.088 Å, and *a* = 4.090 Å for Ag/CS/SiO_2__007, Ag/CS/SiO_2__002, and Ag/CS/SBA-15 samples, respectively) were consistent with the literature data [50,51] (*a*Ag = 4.085 Å) and confirmed the FCC structure of the silver crystal. The full width at half maximum (FWHM) values measured for reflection was used with the Scherrer formula to calculate the average size of the observed silver crystallites [52,53]. Table 1 shows the particle size calculated for several first lattice planes, indexed as (111), (002), (022), and (311), and the textural properties of investigated composites as the porosity properties. In practice, the crystallite size can be calculated in the direction perpendicular to a given plane. The average value of presented values (from four values for each sample) shows the average size of the crystallites presented in the sample and equals 204 Å, 202 Å, and 186 Å for the Ag/CS/SiO_2__007, Ag/CS/SiO_2__002, and Ag/CS/SBA-15 samples, respectively.

The presence of the crystalline phase in the samples was evaluated. Crystallinity refers to the degree of structural order of solids. The crystallinity percentage determined via XRD is obtained by dividing the total area of crystalline peaks by the total area under the diffraction curve (crystalline plus amorphous peaks). The crystallinity percentage is determined via XRD by dividing the total area of crystalline peaks by the total area under the diffraction curve (crystalline plus amorphous peaks). In the present case, the crystallinity of the investigated samples was evaluated. The greatest value for crystallinity was determined for Ag/CS/SBA-15 (84.2%), which means that it had the highest proportion of crystalline phase—and thus the largest amount of metallic nanoparticles—when compared with the other materials (46.9% and 54.6% for Ag/CS/SiO_2__007 and Ag/CS/SiO_2__002, respectively). The degree of crystallinity can also be compared with that of the reference system CS/silica (CS/SBA-15), which had the smallest crystallinity value (30.9%).

Since chitosan fibers obtained in a specific way have an established crystallinity (CS/SBA-15 with 30.9% of crystallinity), the significant changes in crystallinity observed between the investigated samples must be due to the presence of the crystalline phase (AgNP). The increase in the degree of crystallinity and, indirectly, the increase in the amount of metallic phase, was calculated based on the difference in the degree of crystallinity of the investigated sample and the initial sample (without silver nanoparticles, designated as CS/SBA-15). This clearly illustrates that the presence of silver improves the crystallinity and ordering of the chitosan/silica fibers and can be used to evaluate their amount in the sample. Improvement in the degree of crystallinity of composite materials can result from both the presence of silver nanoparticles and an improvement in the order and stability of the fibers themselves in the presence of silver nanoparticles. The observed value of the degree of crystallinity is the sum of both these phenomena. However, an improvement in crystallinity is guaranteed in both cases by the presence of a metallic phase. The TEM images verify the presence of silver nanoparticles in the entire space of the chitosan fibers, which confirms that, during impregnation (i.e., gentle shaking in wet conditions), silver nanoparticles can migrate inside the nanofiber network. Their presence then stabilizes the entire fiber system; therefore, after drying, their reorganization is evident as an increase in crystallinity.

In all cases, the first diffraction peak (Ag(111)) was much more intense and narrower than the others, suggesting different particle sizes depending on the crystal planes. The calculations suggested that the average silver particle size was 10 nm greater for the first lattice plane than for other planes. This trend was preserved for all samples, indicating no visible carrier effect on the morphology of the silver nanoparticles deposited on the nanofibers. 

**Figure 2:**
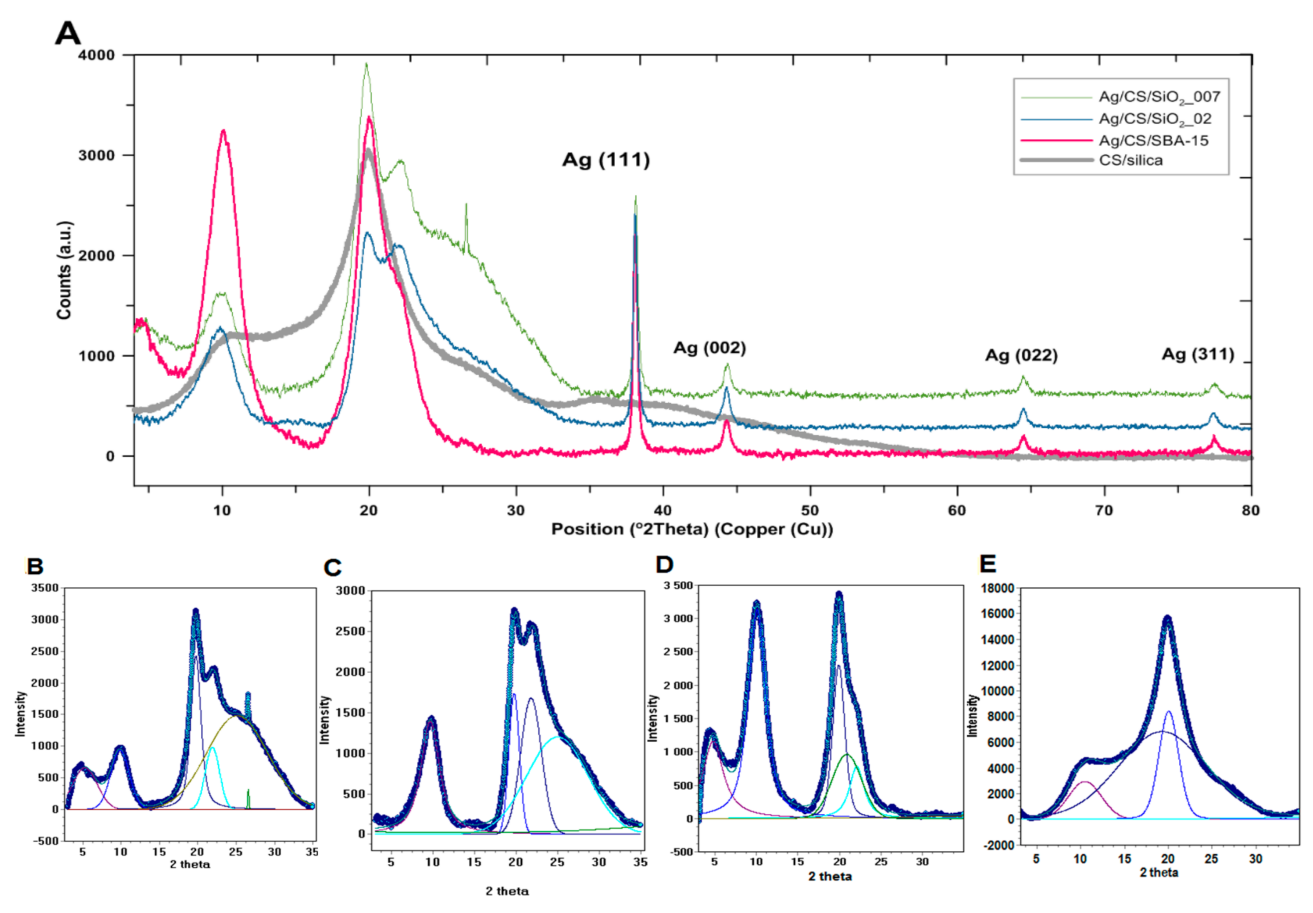
(**A**) X-ray powder diffraction pattern of the Ag/CS/silica (chitosan nanofibers with silica phase decorated by silver nanoparticles) nanofibers, (**B**–**E**) Experimental points and fitted profiles of the observed diffraction patterns (B-Ag/CS/SiO_2__007, C-Ag/CS/SiO_2__02, D-Ag/CS/SBA-15, E-CS/silica (CS/SBA-15).

The XRD patterns in the area of 5–35° of 2θ characterized the crystallinity of the chitosan/silica nanofibers. The obtained XRD profiles shown in Figure 2 suggest that chitosan is not completely crystallized, as a two-phase system is evident in which, apart from the crystalline areas characterized by a spatial arrangement of macromolecules, some amorphous regions typical for semi-crystalline systems are also present. The visible crystallinity (the presence of well-defined XRD patterns) is the effect of the stabilization of the structure and stiffening of the crystalline domains by hydrogen bonds and electrostatic interactions between the N-acetyl groups. Typically, two characteristic crystalline peaks at 10° and 20° of 2θ, with comparable crystallinity related to crystal I and crystal II, respectively, in the chitosan structure [54,55], are distinguished for chitosan, along with minor other reflections at higher 2θ values [56]. 

**Figure 3:**
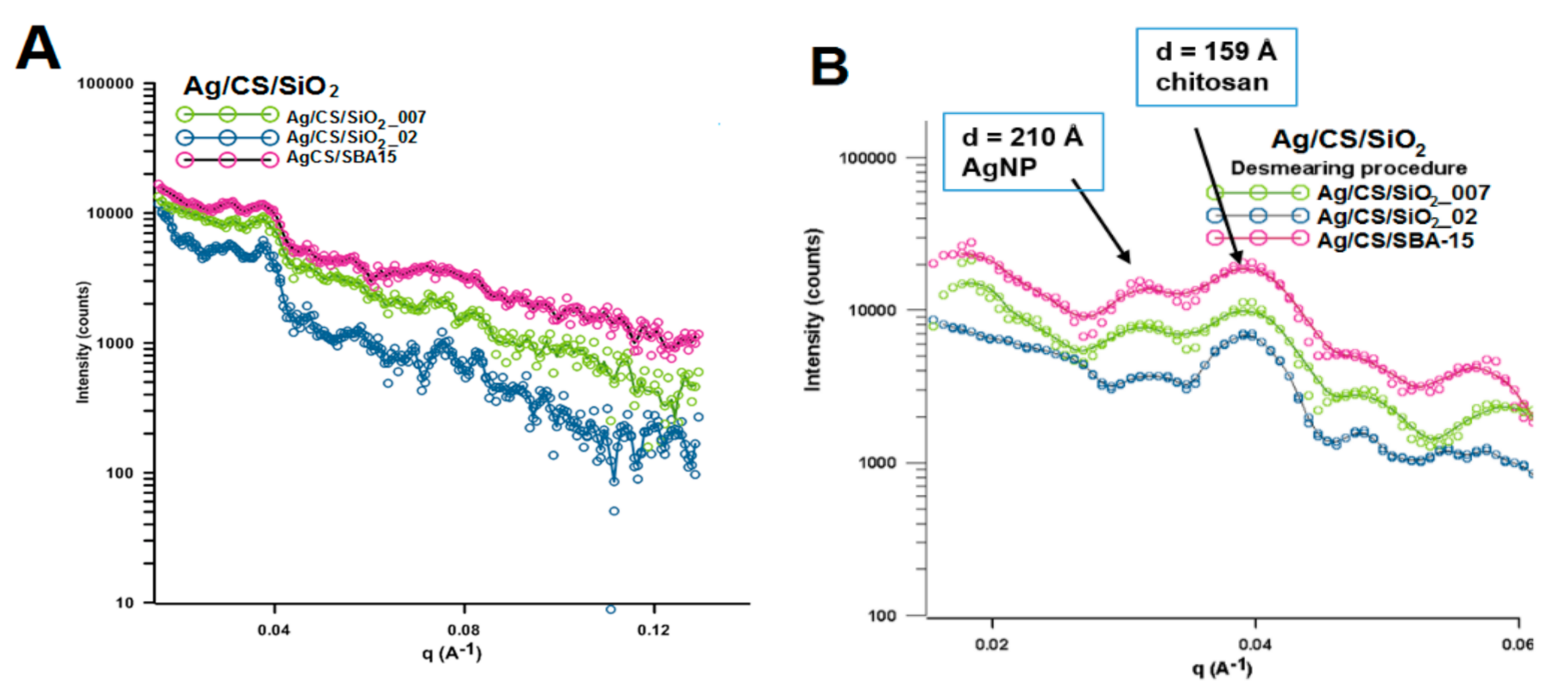
(**A**) Experimental small angle X-ray scattering (SAXS)profiles for Ag/CS/silica composites, (**B**) Experimental SAXS profiles for the investigated samples as line collimation data after desmearing. Note: The desmearing routines in Easy SAXS use a matrix inversion technique based on the Cholesky decomposition.

**Figure 4:**
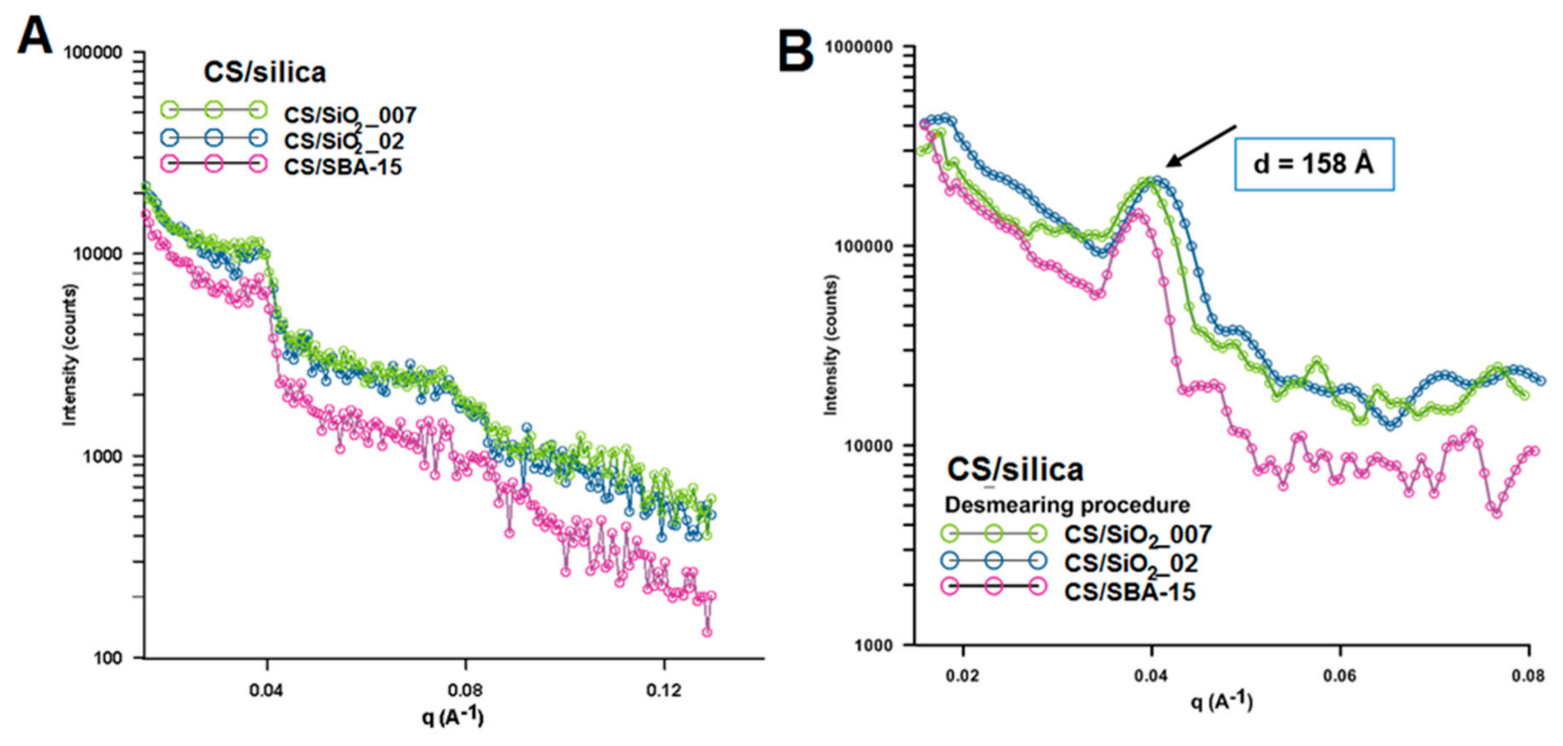
(**A**) Experimental SAXS profiles for CS/silica nanocomposites, (**B**) Experimental SAXS profiles after desmearing.

Using the CS/silica sample (specifically represented by CS/SBA-15 sample) without silver nanoparticles as a reference system, some changes in peak position, peak intensity, and peak width are evident in the area typical for chitosan (from 5–35° of 2θ). These changes are related to the change in the degree of crystallinity between individual samples. Characterization of the morphology of the nanoparticles and their degree of dispersion is important in the development of nanocomposites and was evaluated by SAXS measurements. Figure 3 shows the experimental X-ray scattering data plotted as a function of the scattering vector q. The scattering curves vary in intensity for individual samples. The intensity of the scattering signal is the largest for the Ag/CS/SBA-15 sample and the smallest for Ag/CS/SiO_2__002 (Figure 3A). Taking into account the size of the metallic nanophase (determined from XRD measurements), the sample with the highest level of scattering is the sample with the smallest crystallites. The second level of scattering intensity is noted in sample Ag/CS/SiO_2__007 (with a medial size of crystallites). This reveals a single correlation between the size of the nanophase and X-ray scattering at small angles and may be the first data on the properties of nanoparticles in/on the nanocomposite body. The sizes of electron density inhomogeneity described as:

(1) $$d=\frac{2\pi \;}{q},$$

where *q* = $\frac{2\pi sinƟ}{\lambda }$ were calculated for two peaks on the SAXS curves in the position of *q* = 0.0298 Å^−1^ and *q* = 0.0393 Å^−1^ (Figure 3B). The size of the electron density inhomogeneity d generated by AgNP is noticeable and equals around 210 Å. This may suggest that the size of the silver nanoparticle layer is below 20 nm. After dimensioning the metallic phase by other techniques, this result is fully justified. The calculation performed for the second peak (*q* = 0.0393 Å^−1^) suggests the presence of an explicit area of inhomogeneity d with a size around 16 nm. This observation was clarified by analyzing the SAXS curves for composites without a silver phase (Figure 4). In this case, the experimental curves revealed the presence of only one peak at the position of 0.0394 Å. The calculated d value was very similar (second peak for Ag/CS/silica composites) and almost equaled 16 nm (Figure 4B).

An additional small, but noticeable, shift toward higher values for this signal was observed with increasing grain sizes of the silica phase (SiO_2__007 phase with the average particle size ~7 nm, SiO_2__02 phase with the average particle size ~200 nm, and SBA-15 phase with the average particle size below 1 µm).

Similarly, the intensity of X-ray scattering change according to this dependence (Figure 4A). The higher intensity was observed for CS/SiO_2__007 while the lowest was noticeable for CS/SBA-15. This suggests that the presence of characteristic points on the scattering curve, such as areas with higher intensity, maybe a specific fingerprint of the silica phase and could be useful in the analysis of new composite materials.

#### 2.1.2. Textural Properties of Nanofibers

**Figure 5:**
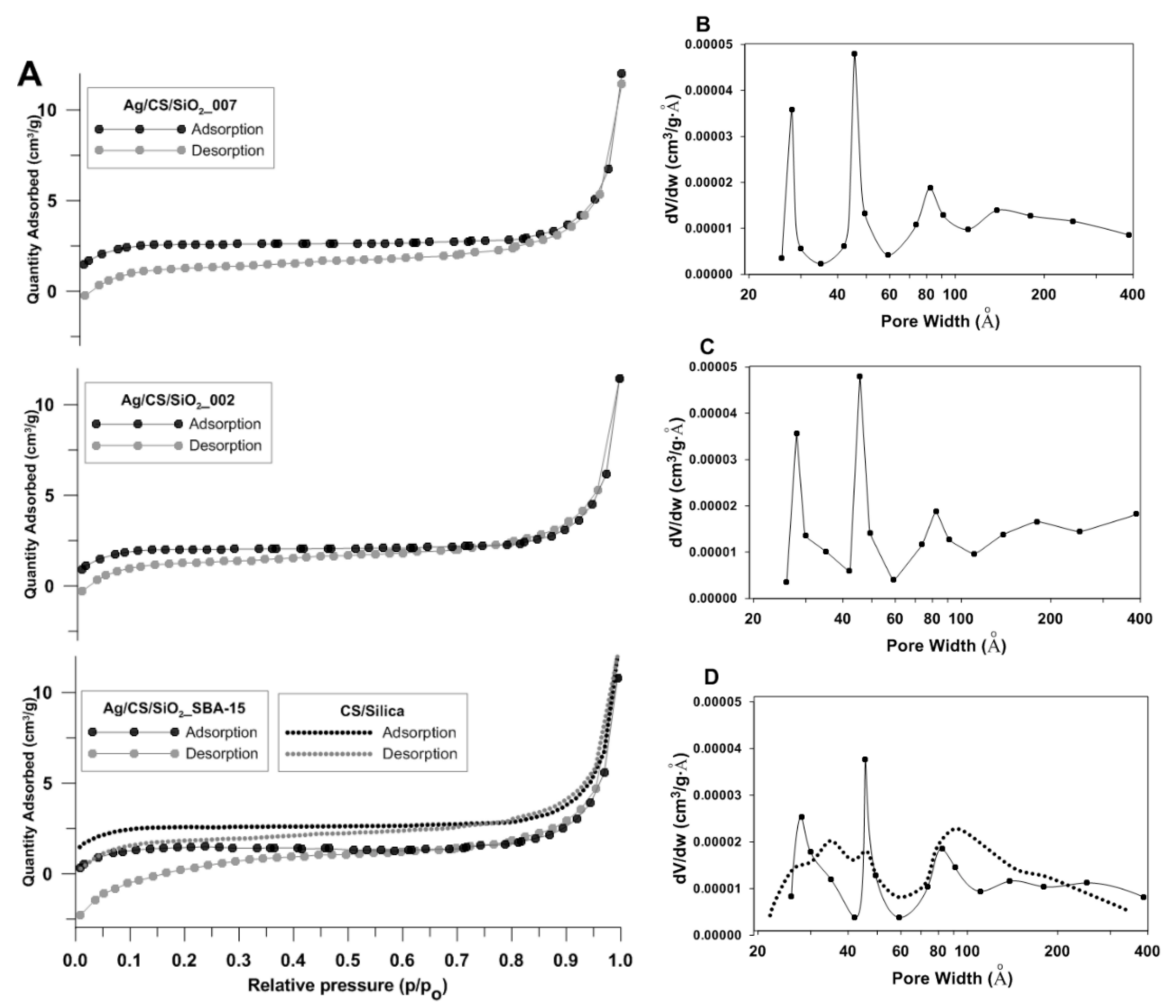
(**A**) Nitrogen adsorption-desorption isotherms plots of Ag/CS/silica nanofiber composites at 77 K. (**B**–**D**) Porosity distributions by the Barrett–Joyner–Halenda (BJH) model were calculated from the adsorption branch of the isotherms. Note: Dotted lines refer to a CS/silica sample used as a reference.

The porous structure of the investigated samples was determined using N_2_ adsorption–desorption analysis at 77 K. The experimental N_2_ gas adsorption-desorption isotherms for the investigated systems, as well as the pore size distributions (PSD) from the desorption data, are presented in Figure 5. Overall, the experimental isotherms are similar in shape for all the investigated samples. The specific surface area of the Ag/CS/silica nanofibers was evaluated at about 8 m^2^/g. The exact values are given in Table 1. In addition to the specific surface area (SBET), Table 1 contains also the values for the total pore volume and pore size distributions as the three most important maxima of the size distribution function PSD (Figure 5B–D). The specific surface area of the CS/silica (CS/SBA-15) nanofibers was also comparable (9.82 m^2^/g). The experimental isotherms of the investigated materials show typical adsorption behaviors for the IV type, according to the IUPAC classification. 

The adsorption branch almost overlapped with the desorption branch, especially when P/P_0_ was greater than 0.8. The very slight presence or the absence of a hysteresis loop below this value indicated opened mesopores without interruption between the capillary condensation and evaporation phenomenon of the nitrogen molecules. Generally, the BET specific surface areas calculated from the isotherms were quite low but similar to results obtained by other research groups. As shown in Figure 5B–D, the investigated composites presented polydispersity of the porous structure with the first, second, and third PSD in the positions of 30, 50, and 80 nm, respectively.

### 2.2. Morphological Studies of Ag/CS/Silica Nanofibers

**Figure 6:**
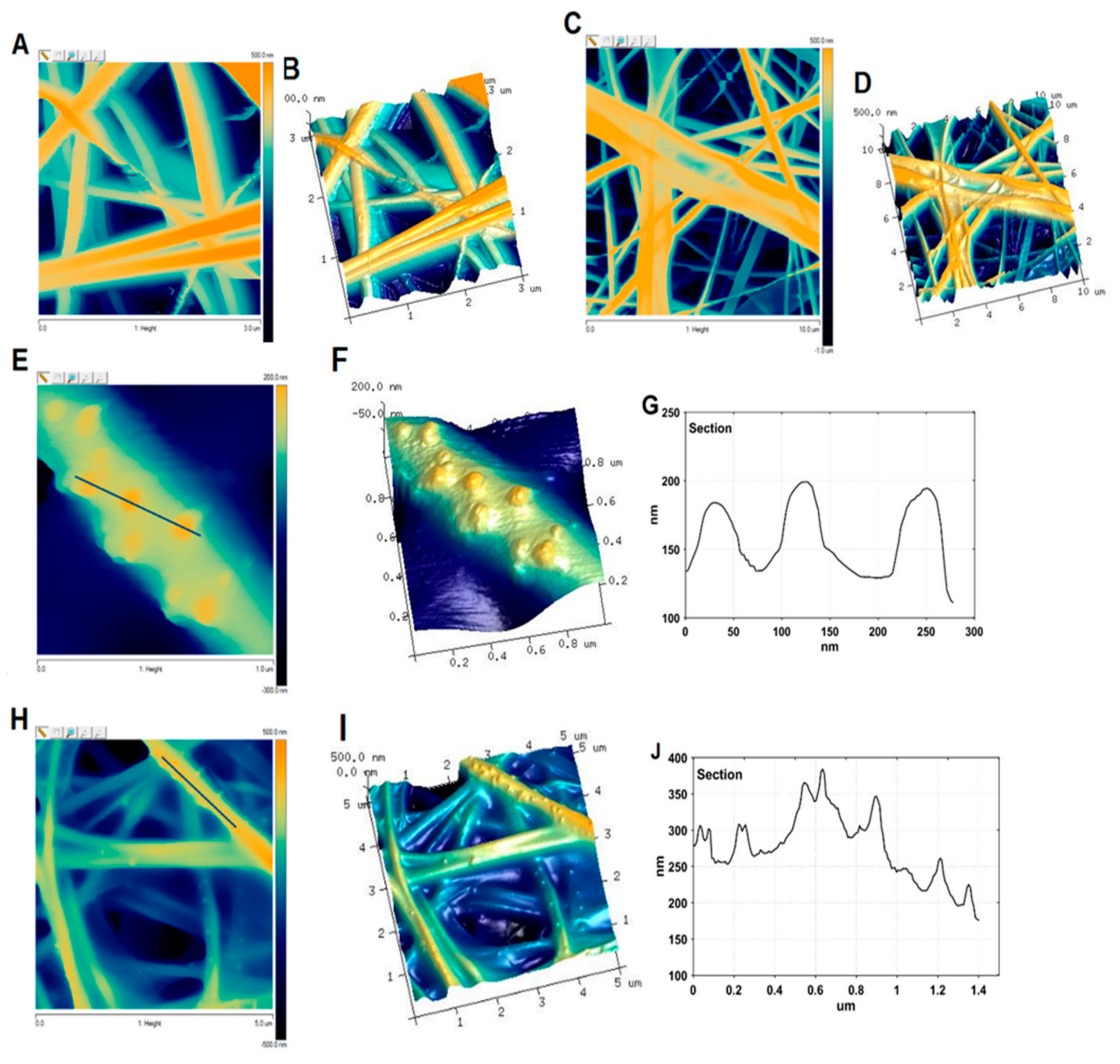
Atomic force microscopy (AFM) topography of the investigated nanofibers. AFM images 3 × 3 µm (**A**) and 10 × 10 µm (**C**) representing the topography of the CS/silica composite and 3D AFM images for these magnifications, respectively (**B**,**D**). AFM images 1 × 1 µm (**E**) and 10 × 10 µm (**H**) of Ag/CS/SBA-15, 3D visualization for these magnifications, respectively (**F**,**I**) and cross-sectional analyses of AFM images (**G**,**J**) according to the lines marked on images (**E**,**H**).

Atomic force microscopy combined with scanning electron microscopy was used as a tool for imaging the Ag/CS/SBA-15 nanofibers, as well as a CS/silica reference sample (CS/SBA-15). Silver nanostructures supported on the nanofibers, as well as the carrier nanofibers, were investigated in this part by estimating their dimensions, nature, and shape. Figure 6 shows the 2D topographies and three-dimensional models of the chitosan/silica (Figure 6A–D) and Ag/CS/SBA-15 (Figure 6E–H) samples, where the surface is reconstructed from the positions of the cantilever. The nanocomposites consist of ultra-fine nanofibers ranging in width from 100 to 300 nm and at least a dozen microns in length.

The surfaces of the electrospun nanofibers without silver nanoparticles (CS/silica) were smooth and continuous (Figure 6A–D). No significant defects, pores, or concrescences were evident on their surfaces. This homogeneous fiber surface changed after incorporation of the metallic nanoparticles, which existed as small, mostly spherical and semi-spherical objects on the surface of the nanofibers (Figure 6E–I). The silver nanoparticles were randomly located on the fibers, without noticeable signs of agglomeration. The size of the AgNPs, determined from AFM images, was estimated based on lines that were marked crossing the visible Ag nanoparticles (Figure 6G,J). The diameters of the AgNPs were 20–50 nm, corresponding to the solid lines marked in Figure 6E,H. The heights of the Ag nanoparticles were also ~50 nm, taking into account the level of nanoscale as the baseline.

**Figure 7:**
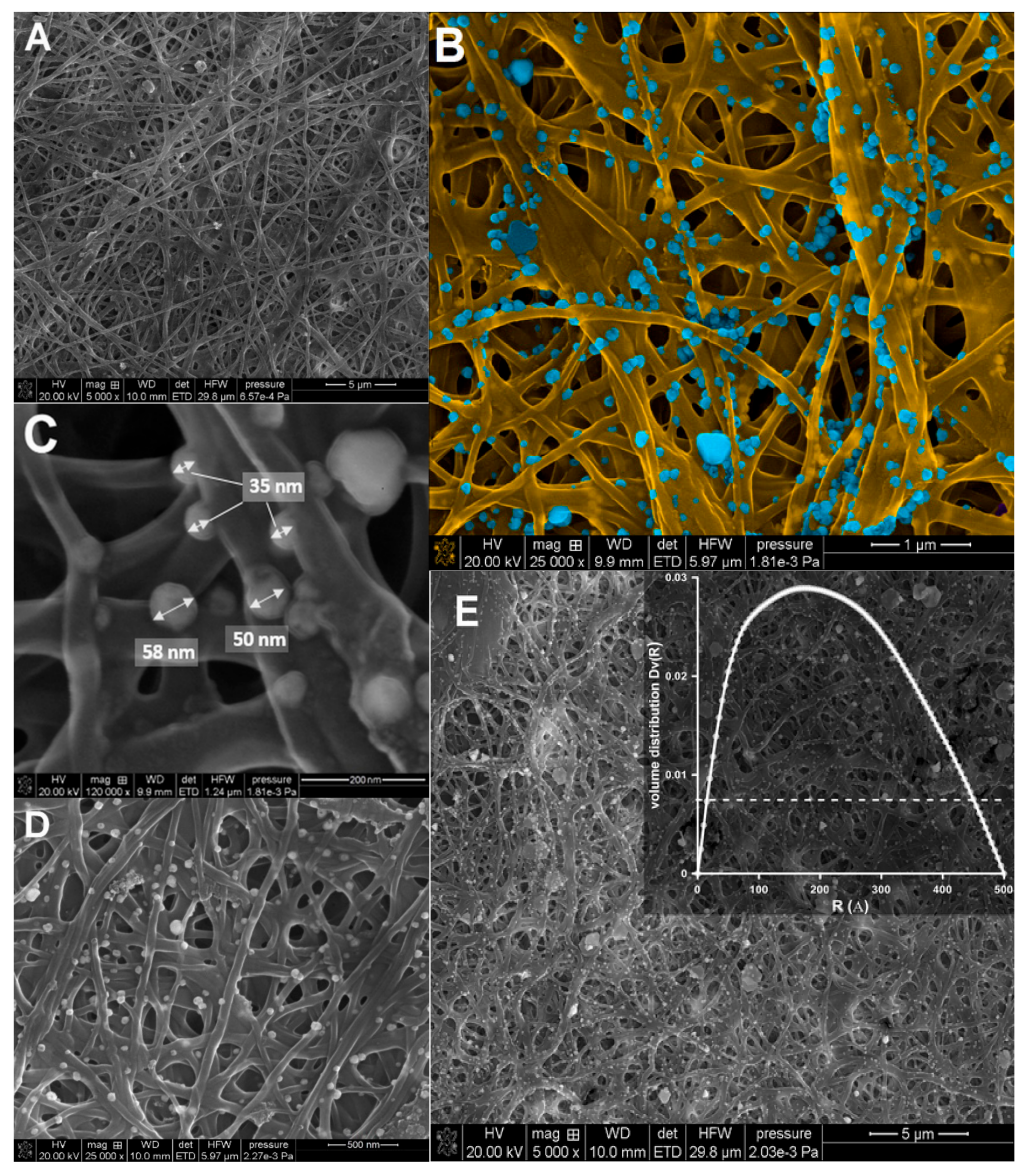
Representative SEM images of the electrospun nanofibers. (**A**) SEM image of CS/silica (CS/SBA-15), (**B**–**E**) SEM images of Ag/CS/SBA-15 at various magnifications. Note: The insert in Figure 4E shows the radial distribution function, Dv(R), obtained from the SAXS data for Ag/CS/SBA-15.

The SEM images of the investigated nanocomposites with silver nanoparticles demonstrated that the AgNPs were homogenously distributed on the CS/silica nanofibers (Figure 7). All the scaffolds were visible in Figure 7 showed a random architecture of the nanofibers, with interconnected pores. The nanofibers formed a three-dimensional network that would be suitable for membrane, filtration, and sorption applications. The sizes of the metallic nanoparticles ranged from 10 to 50 nm, which confirmed our previous observations (XRD and AFM analysis).

The sizes of the nanoparticles estimated from microscopy measurements were also compared to those calculated from SAXS data. The particle size distribution was obtained by analyzing the experimental scattering profiles (Figure 3A). The average diameter of the silver nanoparticles was estimated as above 20 nm, with a maximum distribution of about 20–25 nm. These results agree with the TEM images. Both imaging techniques also suggested that electrospinning produces CS nanofibers with good quality, without beads that act as carriers of nanoparticles; this will enable the creation of composites with a homogeneous distribution of metallic nanoparticles. These results clearly revealed that the addition of a silica phase in a quantity not exceeding 5% does not disturb or negatively affect the morphology of the resulting nanofibers. Although this paper presents only preliminary studies on the introduction of a silica phase into a fiber structure, the obtained results show promise for the possibility of incorporating additional modifications and improvements to generate novel nano-sized fiber structures for various applications.

**Figure 8:**
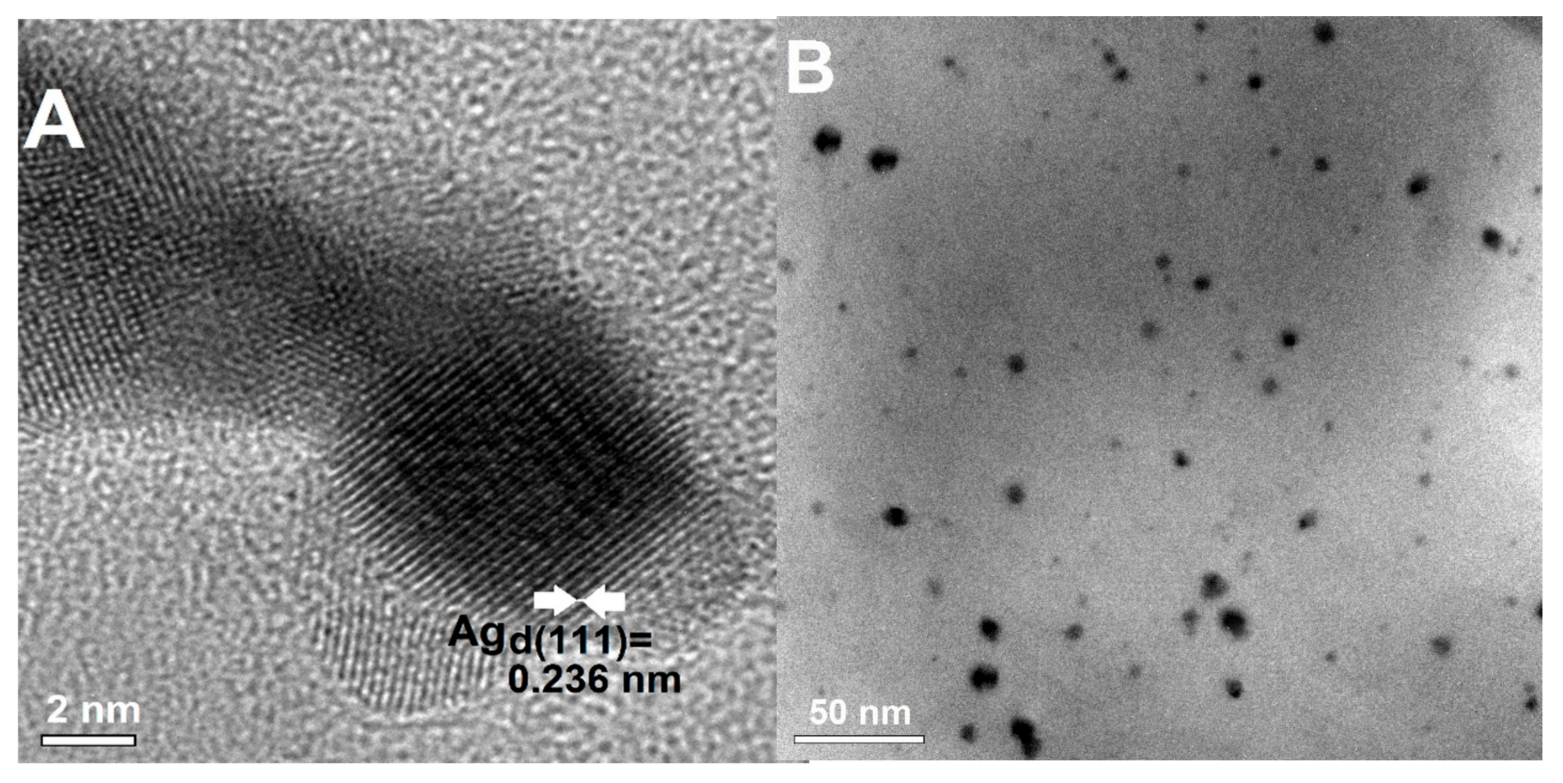
TEM images of the silver nanoparticles extracted from composites. (**A**) The HRTEM image of selected AgNP with visible lattice planes (111) and the interplanar spacing (d111 = 0.236 nm), (**B**) Extracted AgNP in ethanol.

A highly crystalline quality of AgNP on chitosan nanofibers was achieved after their deposition. The morphology and crystallinity of AgNP extracted from composites were evaluated by TEM. Figure 8, which shows the obtained images as a high-resolution image (Figure 8A), illustrates a single AgNP and the general morphology of the extracted nanoparticles at a lower magnification (Figure 8B). Most particles were separated and were nearly spherical, with diameters ranging from 5 to 25 nm. The lattice fringes in the HRTEM image are d = 0.236 nm, which agrees with the first (111) lattice spacing of the fcc Ag phase.

### 2.3. Antibacterial Activity of Ag/CS/Silica Nanocomposites

**Figure 9:**
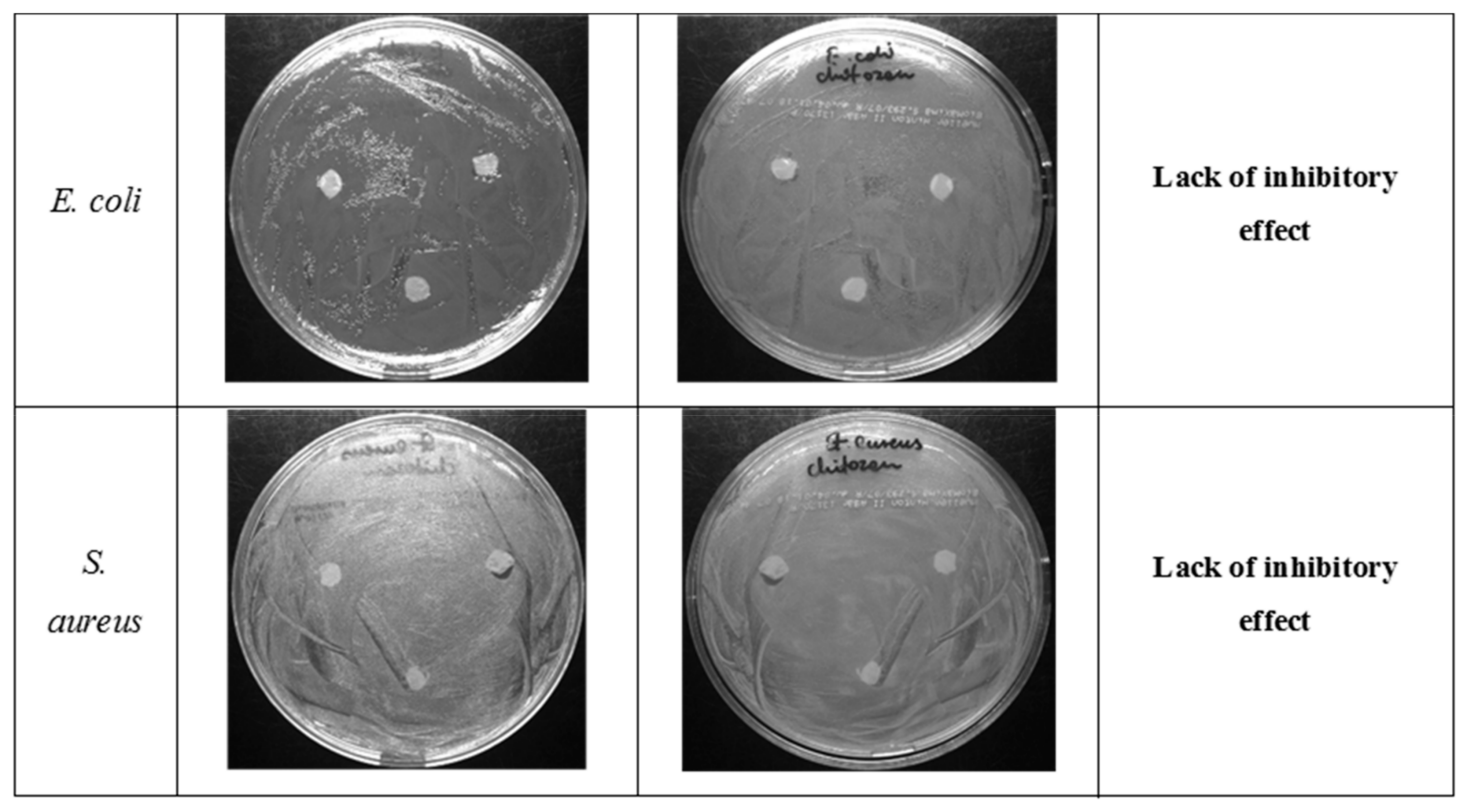
Photographs of the growth of *E. coli* ATCC 25922 and *S. aureus* ATCC 25923 on MH agar plates with placed discs cut from CS/silica nanofibers.

The antibacterial properties of CS/silica (CS/SBA-15), Ag/CS/SBA-15 membranes, and the AgNP solution were determined for *E. coli* ATCC25922 and *S. aureus* ATCC25923 by diffusion tests after a 24 h incubation. In the first stage, the biostatic activity was determined for composites without nano-molecular silver. Neither strain showed a significant zone of inhibited growth around CS/silica (Figure 9). The composites with embedded silver nanoparticles gave quite different results, as clear biostatic activity was observed. The Ag/CS/SBA-15 membrane showed a 10 mm inhibition zone for the *E. coli* ATCC25922 strain and an even larger (13–14 mm) inhibition zone for the *S. aureus* ATCC25923 strain, indicating a slightly higher activity against *S. aureus.*

**Figure 10:**
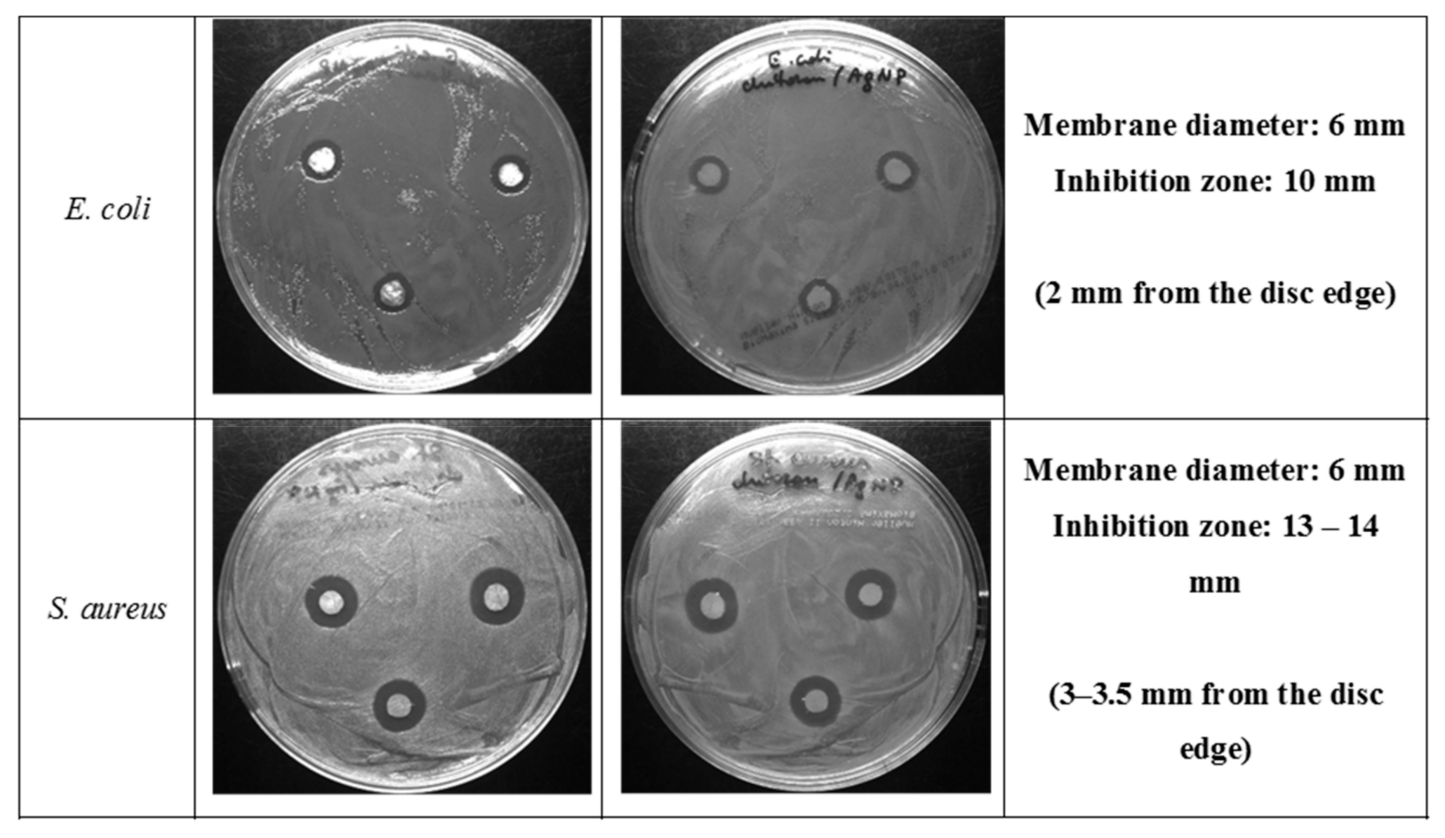
Photographs of the growth of *E. coli* ATCC 25922 and *S. aureus* ATCC 25923 on MH agar plates with placed discs cut from Ag/CS/SBA-15 nanofibers.

The images in Figure 10 show that the inhibition zone around the discs was two-fold larger for the *S. aureus* ATCC25923 strain than for the *E. coli* ATCC 25922 strain. This observation suggests a difference in the antibacterial properties of AgNP on chitosan nanofibers against biofilms formed by Gram-positive and Gram-negative bacteria. A similar effect was observed previously in the literature [57].

**Figure 11:**
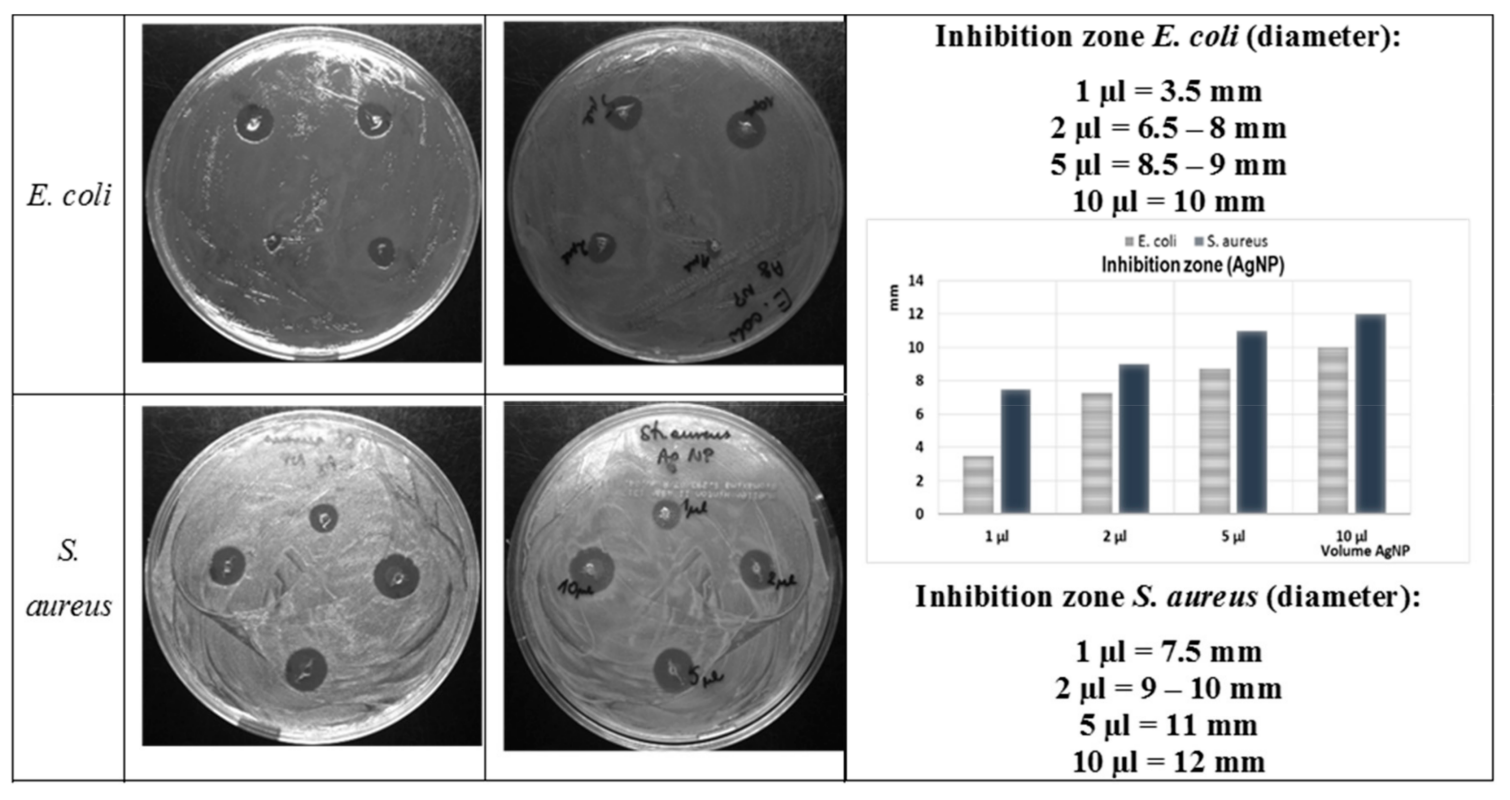
Photographs of the growth of *E. coli* and *S. aureus* on Mueller Hinton agar (MHA) plates with various amounts of AgNP solution and the inhibition zone size (mm) as a function of the AgNP concentration.

The concentration dependence of the antibacterial activity of the AgNP solution was confirmed by comparison studies. The dimensions of the inhibition zones of an AgNP solution (1, 2, 5, and 10 µL) dropped onto the agar medium on which the bacteria were spread, are shown in the images and graph depicted in Figure 11. These results confirmed that AgNP in suspension inhibited the growth of bacteria in a dose-dependent manner. The antimicrobial activity increased with the volume of solution used for both the Gram-positive (*S. aureus* ATCC25923), and the Gram-negative (*E. coli* ATCC25922) bacteria biofilms. However, again, a slightly higher antibacterial activity was observed against the *S. aureus* than against the *E. coli*. 

The antibacterial activity of an AgNP suspension was also evaluated using a microdilution technique. The minimum inhibitory concentration value was 0.68 mg/L for *E. coli* ATCC25922 and two times higher (1.36 mg/L) for *S. aureus* ATCC25923. These results are in good agreement with the data published by Cinteza and co-workers [58] as well as Yuan and co-workers [59]. These results also confirmed the higher tolerance of Gram-positive bacteria to the presence of AgNP in the environment. It should be noted that the sensitivity of the indicator bacteria (*E. coli* and *S. aureus*) used to the tested preparations is comparable and clearly dependent on the form in which the nanosilver was given to the bacterial growth environment.

Bacteria reactions to the presence of nanosilver in their environment are multilevel and depend on so many factors that they cannot be easily predicted and estimated [60]. Silver nanoparticles have high electrical conductivity and cling to the bacterial cell membrane, thereby disrupting the natural gradient of electric potential responsible for the life functions of the bacterial cell. Bacterial membrane permeability, osmoregulation, and electron transport, as well as respiration processes, can be impaired by AgNP. Nanosilver can also interact with cytoplasmic components after its entry into the cells. AgNPs are confirmed to induce oxidative stress that leads to the death of bacteria. Silver ions (released from the AgNP surface) also deactivate bacterial proteins by binding with thiol (-SH) groups on the proteins. The presence of a significantly thicker layer of peptidoglycan in Gram-positive bacteria creates a greater chance of survival for these species [58,61,62]. The results presented here also suggest that the antibacterial activity can be improved by the presence of the chitosan phase and indicate the possibility of a better release of Ag+ ions that can be achieved using AgNP alone [63]. Comparison of the activity of the same amount of AgNP (1 µL of AgNP dropped on the composite discs and 1 µL used directly) showed a significantly better activity of the silver nanoparticles when stabilized on the chitosan structure, thereby confirming the synergy resulting from the combination of a biopolymer and silver nanoparticles.

## 3. Materials and Methods

### 3.1. Reagents

Crab chitosan (MW 110,000; the degree of deacetylation 0.74), was purchased from Sigma-Aldrich. Polyethylene oxide (PEO) (MW 200,000), silver nitrate (≥99.0%), and polyvinylpyrrolidone (PVP) (MW 10,000) were also purchased from Sigma-Aldrich. Fumed silica materials with the particle size of 0.2 µm and 0.007 µm marked as SiO_2__02 and SiO_2__007 were obtained from Sigma-Aldrich. The mesoporous ordered silica SBA-15 was prepared on the basis of the procedure described in detail in our previous work [64]. Aqueous acetic acid (≥99.7%) was obtained from POCH (Avantor Performance Materials Poland S.A). Two bacterial strains, *Escherichia coli* ATCC 25922 and *Staphylococcus aureus* ATCC 25923, were chosen for testing antibacterial activity. All solutions were prepared with deionized water, and reagents were used as supplied, without further purification.

### 3.2. Preparation of CS/Silica Nanocomposites by Electrospinning

Chitosan solution was prepared by dissolving 2 g of polymer in 70 mL of 70% acetic acid with magnetic stirring at room temperature until a clear solution was obtained. A stable solution of colloidal silica (0.1 g of silica in 20 mL of water) was then added to the chitosan solution. The silica content in the final solutions did not exceed 5%. Finally, 6.68 mL of a 3% PEO solution (10% relative to chitosan) was added to the solution and stirred continuously at room temperature until a homogenous solution was obtained. 

The electrospinning procedure was carried out using a laboratory electrospinning equipment NS LAB 500 (Elmarco, Liberec Czech Republic). The polymer solutions were placed in a Teflon cylinder with an active electrode in a parallel position to the collecting electrode. The parameters of the process were adjusted as follows: the distance from the active electrode to the collecting electrode was 15 cm, the driving voltage was 76 kV, and the electrode speed was 16 rpm. The nanofibers were collected on a layer of paper. The final electrospun materials were dried at room temperature overnight and given a final thermal treatment at 80 °C for 5 h. The obtained CS/silica nanocomposites were marked as CS/SiO_2__02, CS/SiO_2__007, and CS/SBA-15 depending on the silica type.

### 3.3. Preparation of the Diammine Silver Precursor and the Colloidal Silver Solution (AgNP)

Diammine silver complex [Ag(NH_3_)_2_]^+^ was prepared by the addition of sodium hydroxide (1.25 M) to silver nitrate (0.3 M). A solid precipitate of silver(I) oxide was immediately produced, and 1 g of this precipitate was separated from the solution and dissolved completely in concentrated (25%) ammonium hydroxide (2.63 mL) to yield a transparent solution of diammine silver complex. The chemical reactions in this step were described in detail in our previous work [64]. Silver nanoparticles (AgNPs) were prepared by chemical reduction of a [Ag(NH_3_)_2_]^+^ complex using formaldehyde as a reducing agent and PVP polymer as a stabilizer. A 5 g sample of PVP was completely dissolved in 50 mL H_2_O and the desired amount of silver diammine solution was added with stirring. Two drops of formaldehyde were then added to the silver-stabilizer solution to generate the silver colloidal solution. The formation of silver nanoparticles was indicated by a change in the visual appearance of the solution from colorless to yellow-green and by the surface Plasmon effect visible in the UV-Vis spectrum. The silver concentration was determined as 43.5 mg/L by atomic absorption spectrometry.

### 3.4. Preparation of the Ag/CS/silica Composites

Square pieces (2 × 2 cm) of suitable CS/silica materials were immersed in 25 mL of freshly prepared colloidal silver solution in closed glass bottles and shaken for 48 h at 25 °C in an incubator. The samples were then gently removed from the solution and air dried at room temperature. The final materials were marked as Ag/CS/SiO_2__02, Ag/CS/SiO_2__007 and Ag/CS/SBA-15. These Ag/chitosan/silica composites were used for structural, textural, and morphological characterization.

### 3.5. Antibacterial Activity Assay and Minimum Inhibitory Concentration (MIC) of AgNP Solution Determination

The antibacterial activities of Ag/CS/silica (specifically for Ag/CS/SBA-15) and AgNP solution were investigated by a zone inhibition method. *S. aureus* ATCC 25923, and *E. coli* (ATCC 25922) were used as indicator strains. These bacterial cultures were maintained on Mueller-Hinton medium (MH broth or MH agar, BioMaxima, Lublin, Poland). The membranes were cut into 6 mm diameter discs and the AgNP solution (from 1 to 10 µL of AgNPs stock solution containing 43.5 mg Ag/L) was applied directly into 2 mm diameter wells made in MH agar poured on Petri dishes. Petri dishes with MH agar were inoculated with 100 µL of bacterial cultures (suspensions containing around 1 × 108 colony forming units/mL). Membrane discs (3 pieces per plate) were transferred onto the dishes containing bacteria and incubated for 24 h at 37 °C (*E. coli*) and at 32 °C (*S. aureus*), respectively. The antibacterial activity was determined by measuring the diameter of the inhibition growth zone around each disk or well. 

The minimum inhibitory concentration (MIC) was determined using 96-well round-bottom microtiter plates. The wells were filled with 100 µL MH broth, 100 µL of stock AgNP solution was added to the first well of each row of wells, and the contents were thoroughly mixed. Two-fold serial dilutions were then completed to the twelfth well and the 100 μL mixture from the last well was discarded. Next, 10 μL of diluted bacterial suspension (5 × 104 cell/mL) was added to all wells (except for the wells used as a broth sterility control). After overnight (20 h) incubation at 37 °C (*E. coli*) and at 32 °C (*S. aureus*), the optical density at 600 nm was measured (automatic plate reader, ASYS UVM340, Biogenet, Józefów, Poland). The MIC value was the lowest concentration that prevented the growth of microorganisms. Microdilution experiments were performed in triplicate for each bacterial species and average MIC values were presented.

### 3.6. Measurements and Calculations

The obtained Ag/CS/silica nanofiber composites were characterized by X-ray diffraction (XRD) with an Empyrean diffractometer (PANalytical) and CuKα radiation (λ = 1.5418 Å) in the range of diffraction angles from 10 to 70 degrees of 2θ. The X-ray diffraction analysis system was equipped with a 9430 033 73104 Cu LFF HR X-ray tube with generator settings of 40 kV and 30 mA (during analysis) and a PIXcel3D detector used in a scanning line 1D detector mode. The measurements were performed using a reflection-transmission spinner suitable for powders and solid objects in reflection geometry. The crystallite size was determined from the full width at half maximum of the X-ray diffraction peaks by Scherrer theory based on the width of the X-ray lines [65].

The nanocrystallite size *L* was calculated as a function of the peak width using the Scherrer equation:

(2) $$L=\frac{k\lambda }{\beta cos\theta },$$

where *k* is a constant related to crystallite shape (*k* = 0.9), *λ* is the X-ray wavelength and equals 1.5418 Å, *θ* is the peak position in radians, and *β* is the full width at half maximum of the peaks located at any 2θ position.

The fitting of the experimental patterns was performed using WAXSFIT software [66]. The corresponding lattice constant was calculated theoretically with the formula *a* = dhkl·$\sqrt{h^{2}+k^{2}+l^{2}}$. 

AFM analysis was performed on a Bruker-Veeco-Digital Instruments Multi-Mode Atomic Force Microscope. For the mapping topography, the tapping mode consisted of tapping the surface with an oscillating probe. For each sample, several regions were chosen for the investigation to obtain more representative results. The measurements were conducted using an antimony n-doped Si cantilever TAP150A with a force constant k = 5 N/m and resonance frequency f0 = 150 kHz. The NanoScope Analysis software from Bruker was applied for the analysis of the AFM data. Atomic absorption spectrophotometry with a graphite furnace (GFAAS) was applied for the determination of initial and final silver concentration in AgNP solutions.

Transmission electron micrographs were obtained with a Titan G2 60-300 (FEI) instrument operating at 200 kV. Scanning electron micrographs were collected from Quanta 3D FEG (FEI). Transmission electron microscopy (Tecnai G2 T20 X-TWIN) was used to record AgNP size and morphology after their extraction from the solid phase. For this study, the samples were prepared by placing a drop of AgNP extracted from the AgNP/CS composite onto a carbon-coated copper grid, drying in the air, and then transferring the grid to the microscope operated at an accelerated voltage of 200 kV

Textural properties of the initial and functionalized materials were obtained by measuring N_2_ adsorption/desorption isotherms at −196 °C over the full range of relative pressures, using a Micromeritics ASAP2020 instrument. Specific surface areas (SBET) were estimated from experimental isotherms by applying the Brunauer–Emmett–Teller (BET) theory. Pore-size distribution curves were obtained from the adsorption branch using the Barrett–Joyner–Halenda (BJH) model with cylindrical pores. All samples were outgassed at 120 °C for 24 h in the degassing port of the analyzer prior to analysis. The localized surface plasmon resonance properties of undeposited AgNP were determined by UV–Vis spectrometry using a UV-Vis spectrophotometer Cary 4000 (Varian Inc.) and a quartz cuvette.

## 4. Conclusions

In the world of nanocomposites, the addition of nanofillers in the form of well-defined nanoparticles to a polymer matrix allows tailoring of their physicochemical properties. The addition of noble metal-based nanoparticles onto polymer nanofibers is a versatile route for fabricating new biocide materials and further extending the wide range of applications of nanocomposites. The biocidal properties of Ag/CS/silica composites were confirmed, whereas the CS/silica composite shows rather inhibited cell proliferation properties. The minimum inhibitory concentration value estimated for *E. coli* ATCC25922 was 0.68 mg/L and twice higher (1.36 mg/L) in case of *S. aureus* ATCC25923. These results suggest the higher tolerance of Gram-positive bacteria to the presence of nanosilver particles. Because the reproducibility and comparability of bioactive composite formation techniques should also be taken into account, in this work we present the complete and simple route from synthesis to the determination of the activity of AgNP-containing chitosan nanocomposites. The obtained composites were characterized by a diffraction method, with particular emphasis on microstructural and microscopy studies. An additional phase in the composite structure (for example, a silica phase), which can improve the mechanical properties and stability of chitosan composites, can be easily interpreted by SAXS measurements. The same measurement also provides information about the metallic phase, by determining the size of the electron nonuniformity. Materials with an even distribution of metallic nanoparticles have been obtained in the present work. Small variations in the dimensions of the nanoparticles (20–50 nm) guarantee their biostatic activity.

## Figures and Tables

**Table 1:** The values of textural parameters describing the porous structure of the samples.

Sample	The Particle Size of Ag Nanoparticles from XRD Data L (Å)				Crystallinity (%)	Surface Area (SBET)^1^ (m2/g)	Pore Volume^2^ (cm3/g)	Pore Size Distribution^3^ (nm)
	Diffraction Peaks (hkl)							
	111	002	022	311				
Ag/CS/SiO_2__007	266	161	195	197	46.9	8.5	0.018	2.8, 4.5, 7.8
Ag/CS/SiO_2__002	281	175	178	175	54.6	8.1	0.018	3.0, 4.6, 8.0
Ag/CS/SBA-15	252	163	165	165	84.2	7.8	0.017	3.0, 4.6, 8.1

^1^ BET surface area, calculated using experimental points at relative pressures of (P/P0) 0.035–0.31, where P and P0 denote the equilibrium and saturation pressures of nitrogen, respectively; ^2^ Total pore volume calculated from the nitrogen volume adsorbed at P/P0 = 0.99; ^3^ Pore-size distribution curves were obtained from the adsorption branch using the Barrett–Joyner–Halenda (BJH) model with cylindrical pores.
